# Profiling type I and II interferon responses reveals distinct subgroups of pediatric patients with autoinflammatory disorders

**DOI:** 10.1016/j.jacig.2025.100450

**Published:** 2025-03-08

**Authors:** Anaïs Nombel, Magali Perret, Sophie Trouillet-Assant, Marine Villard, Christine Lombard, Lorna Garnier, Anne-Perrine Foray, Sarah Benezech, Remi Pescarmona, Samira Khaldi-Plassart, Thierry Walzer, Alexandre Belot, Sébastien Viel

**Affiliations:** a Immunology Laboratory, Hospices Civils de Lyon, Lyon Sud Hospital, Lyon, France; b The International Center of Research in Infectiology, Lyon University, INSERM U1111, CNRS UMR 5308, ENS, UCBL, Lyon, France; c Joint Research Unit Hospices Civils de Lyon-bioMérieux, Lyon Sud Hospital, Pierre-Bénite, France; d Institut d’Hématologie et Oncologie Pédiatrique, Centre Léon Bérard, Lyon, France; e Immunology Laboratory, Hospices Civils de Lyon, Edouard Herriot Hospital, Lyon, France; f National Referee Centre for Rheumatic and Autoimmune Diseases in Children, RAISE, Paris and Lyon, France; g Pediatric Nephrology, Rheumatology, and Dermatology Department, Hospices Civils de Lyon, Hôpital Femme Mère Enfant, Bron, France; h European Reference Network on Immunodeficiency, Autoinflammatory and Autoimmune Diseases (ERN-RITA); i Biotherapy and ATMP Production Platform, Hospices Civils de Lyon, Edouard Herriot Hospital, Lyon, France

**Keywords:** Interferonopathy, IFN signature, NanoString, Simoa, type I interferon, type II interferon, lupus, juvenile idiopathic arthritis

## Abstract

**Background:**

Elevation of type I interferon (IFN-I) is characteristic of a group of diseases known as type I interferonopathies. Several technologies are available to monitor IFN-I, but there is no consensus on their routine use in medical laboratories.

**Objective:**

We aimed to compare the performance of two technologies for this purpose: NanoString, which monitors messenger RNA expression of interferon-stimulated genes (ISGs), and Simoa, which quantifies IFN-α2 protein in an ultrasensitive way. We also designed a NanoString assay to monitor type II ISGs and tested its value to discriminate clinical conditions.

**Methods:**

A total of 196 samples from patients with diseases associated or not with IFN-I pathway activation were analyzed by NanoString and Simoa.

**Results:**

The comparison between NanoString IFN-I score and IFN-α2 Simoa revealed a *r*^2^ coefficient of 0.55. We identified *IFI27, IFI44L,* and *SIGLEC1* as the ISGs most closely related to IFN-α2 concentration. Nineteen samples had a positive IFN-I score but undetectable IFN-α2. These samples were also positive according to IFN-II score, pointing to IFN-II as the primary ISG inducer in corresponding patients. By measuring IFN-I and IFN-II scores in a subset of patients with systemic lupus erythematosus and systemic juvenile idiopathic arthritis, we identified two subgroups of patients in whom IFN-I and IFN-II were dominant.

**Conclusion:**

Both IFN-α2 quantification and NanoString reliably distinguish type I interferonopathies from other diseases. Type I and II interferons induce different transcriptomic signatures *in vitro* and *in vivo,* and our results highlight the value of monitoring both IFN-I and IFN-II in interferon-related diseases.

The interferon system comprises multiple types: type I (IFN-I), type II (IFN-II), and type III (IFN-III). The IFN-I family includes 13 IFN-α subtypes, IFN-β, IFN-ε, IFN-κ, and IFN-ω in humans. IFN-Is are produced following the engagement of innate sensors located at the cell surface, in the endosomes or in the cytoplasm. On activation, sensors signal through different transduction pathways that converge toward the activation of transcription factors, leading to the production of IFN-I.^1^ Newly produced IFN-I bind to their receptor, IFN-α/β receptor (IFNAR), and act in an autocrine and paracrine way via the IFNAR/Janus kinase (JAK)/STAT (signal transducer and activator of transcription) pathway, initiating the transcription of hundreds of interferon-stimulated genes (ISGs). These ISGs play a prominent role in the acute immune response against viruses at multiple levels as they encode antiviral proteins and other proteins regulating the immune response. Despite having distinct receptors, IFN-I, IFN-II, and IFN-III share common signaling pathways (eg, JAK/STAT pathway), leading to overlapping ISG patterns.^1^, ^2^, ^3^

While the acute expression of IFN-I at early phases of viral infection protects from severe disease, an aberrant and chronic expression of IFN-I causes a group of Mendelian diseases called interferonopathies.^1^^,^^2^^,^^4^^,^^5^ These diseases are either due to mutations in genes involved in nucleic acid degradation or to gain-of-function mutations in genes involved in the IFN-I pathway, and the hallmark of these diseases is an elevation of blood IFN-α. A high level of blood IFN-α concentration is also characteristic of autoimmune diseases like dermatomyositis (DM), scleroderma, and systemic lupus erythematosus (SLE).^6^, ^7^, ^8^ In SLE, the systemic level of IFN-α is used as biomarker of disease activity and identifies patients at risk of relapse during remission, making drugs targeting this pathway promising therapeutic options. In DM, ISG expression signatures also correlate with disease activity.^9^, ^10^, ^11^, ^12^, ^13^, ^14^, ^15^, ^16^

Given the central role of IFN-I in these pathologies, several assays have been developed to explore IFN-I pathway activation. Existing assays measure the activation of the IFN-I pathway at different levels (interferon proteins, interferon-induced proteins, messenger RNA [mRNA] expression of ISGs, functional assays) and using several technologies.^17^^,^^18^ Quantifying IFN-I at the protein level is a challenge because it circulates at very low levels, but the recent development of single molecule array digital ELISA (Simoa) now enables the detection of blood IFN-α2 protein.^9^^,^^10^^,^^19^ Several groups also developed indirect assays measuring the expression of a set of ISGs at the mRNA level and calculated an ISG expression score reflecting IFN-I pathway activation.^20^^,^^21^ According to the laboratories, the two approaches have been used, but data comparing the techniques are missing in a clinical setting, and there is currently no consensus on the biological tools to use in routine clinical practice.^18^^,^^22^^,^^23^

A type I interferon monitoring assay, hereafter referred to as the IFN-I score, has been set up in routine practice in our laboratory in Lyon since 2018.^24^ This IFN-I score consists of a score calculated on the basis of the mRNA expression level of 6 ISGs using NanoString nCounter technology (NanoString Technologies, Seattle, Wash). The aim of this study was to evaluate the diagnostic performance of the IFN-I score compared to IFN-α2 quantification by Simoa in a large cohort of pediatric patients. Because some patients had a high IFN-I score but low IFN-α2 levels, we set up a NanoString-based IFN-II score to explore both type I and type II ISGs, allowing us to discriminate between patients exposed to either of the two interferons.

## Methods

### Participants

This study was carried out in children hospitalized at the Hospices Civils de Lyon between 2017 and 2018, whose disease was classified using international classifications into monogenic type I interferonopathies, SLE, DM, mixed connective tissue diseases (mCTDs), systemic juvenile idiopathic arthritis (sJIA), and macrophage activation syndrome (MAS) (Table I). The study was approved by the ethics committee of the Hospices Civils de Lyon (Lyon University Hospital approval 23-5124) for the protection of the study participants and was conducted in accordance with the Declaration of Helsinki ethical principles and following good clinical practices.

### ISG expression signatures

#### IFN-I signature

The NanoString-based protocol for the interferon signature was previously published.^24^^,^^25^ The expression of 6 ISGs—*SIGLEC1* (sialic acid binding immunoglobulin-like lectin 1), *IFI27* (IFN-α–inducible protein 27), *IFI44L* (interferon-induced protein 44–like), *IFIT1* (interferon-induced protein with tetratricopeptide repeats 1), *ISG15* (interferon-stimulated gene 15), and *RSAD2* (radical *S*-adenosyl methionine domain containing 2)—was measured for IFN-I score (Table II).

**Table I:** Characteristics of study population

Characteristic	SLE	DM	mCTD	Interferonopathy	Other diseases∗
No. of subjects	22	7	9	4	74
Sex, F/M	20/2	5/2	6/3	2/2	35/39
Age (years), mean ± SD	14.9 ± 2	11.7 ± 3.7	14.4 ± 2.3	13.1 ± 2.8	10.5 ± 5.0
Active disease	12 (55)	4 (80)	5 (56)	0	NP
Treatment in month before sampling					
Corticosteroids	7 (32)	3 (43)	5 (56)	0	14 (19)
Hydroxychloroquine	16 (73)	2 (29)	5 (56)	1 (25)	0
Rituximab	2 (9)	0	0	0	1 (1)
Methotrexate	1 (5)	4 (57)	2 (22)	0	3 (4)
AZT, MMF, leflunomide, tacrolimus, everolimus, cyclosporine, thalidomide	8 (36)	0	4 (44)	0	3 (4)
Anti–TNF-α	1 (5)	0	0	0	3 (4)
JAK inhibitor				1 (25)	
Other treatment†				0	15 (20)
No treatment	3 (14)	1 (14)	3 (33)	3 (75)	39 (53)
IFN-I score, median (min-max)	45.44 (3.56-84.21)	21.04 (3.19-80.7)	31.25 (12.3-67.81)	39.97 (21.54-56.13)	4.1 (<0.4-38.87)
IFN-α2 (fg/mL), median (min-max)	157 (<3.2-25790)	13.4 (<3.2-108)	522 (<3.2-38630)	297 (78.7-6812)	8.1 (<3.2-1477)

Data are presented as nos. (%) unless otherwise indicated.

*AZT,* Azathioprine; *MMF,* mycophenolate mofetil; *NP*, not provided.

∗ Included children hospitalized at the Hospices Civils de Lyon in pediatric nephrology, rheumatology, dermatology, and neurology departments who were diagnosed with diseases not known to be mediated by IFN-I.

† Included tocilizumab, anakinra, canakinumab, colchicine, and intravenous immunoglobulins.

#### IFN-II signature

Using the NanoString-based protocol,^24^^,^^25^ we measured the expression of 21 IFN-γ–induced genes previously identified^26^, ^27^, ^28^ (see Table E1 in the Online Repository available at www.jaci-global.org) at the mRNA level in whole blood samples from healthy donors (HDs) at baseline and after 24 hours’ stimulation with IFN-α or IFN-γ. We selected the 5 ISGs most strongly induced by IFN-γ but minimally or not at all by IFN-α: *ANKRD22* (ankyrin repeat domain 22), *FCGR1B* (Fc gamma receptor Ib), *HLADMB, HLADPB1,* and *HLADRB3* (Table III). The expression of these ISGs was used to define the IFN-II signature.

**Table II:** Signature IFN-I probes used for ISG and housekeeping gene quantification with NanoString technology

Gene	Accession no.	Probe	Sequence (5′-3′)
*SIGLEC1*	NM 023068.3	A	CAACACTGCCTCATTCACATTCATAGGCTGGAGTCATCACAGATTCTGCGAACCTAACTCCTCGCTACATTCCTATTGTTTTC
		B	CGAAAGCCATGACCTCCGATCACTCCATAAAAAGTCAGATGTCACAGAGCTGTTTTCGTAGAGGCGGGCAGGACT
*IFI27*	NM 005532.3	A	GAGCCCAGGATGAACTTGGTCAATCCGGAGAGTCCAGTTGCCTGTTGAGATTATTGAGCTTCATCATGACCAGAAG
		B	CGAAAGCCATGACCTCCGATCACTCGGAGCTAGTAGAACCTCGCAATGACAGCCGCAATGGCAGACCCAATG
*IFI44L*	NM 006820.2	A	CTTCTGCCCCATCTAGCCCCATAGTGTCACACAACATAAATGGCAGAGATCAAAGACGCCTATCTTCCAGTTTGATCGGGAAACT
		B	CGAAAGCCATGACCTCCGATCACTCCATACAACCTTTTAAGATGTGGGGAATGTCATCCATGCACAGTCCTGCTC
*IFIT1*	NM 001548.3	A	TGTAGACGAACCCAAGGAGGCTCAAGCTTTCCAGATCTAATGCCTTTCTCCCAATTTGGTTTTACTCCCCTCGATTATGCGGAGT
		B	CGAAAGCCATGACCTCCGATCACTCCAGGGCCCGCTCATAGTACTCCAGGGCTTCATTCATATTTCCTTCCAATT
*ISG15*	NM 005101.3	A	CTCAGAGGTTCGTCGCATTTGTCCACCACCAGCAGGACCTTTCGGGTTATATCTATCATTTACTTGACACCCT
		B	CGAAAGCCATGACCTCCGATCACTCTGCTGCTGCGGCCCTTGTTATTCCTCACCAGGATG
*RSAD2*	NM 080657.4	A	CCGTCCCTTTCTACAGTTCAGAAAGCGCATATATTCATCCAGAATAAGGTCAACAGCCACTTTTTTTCCAAATTTTGCAAGAGCC
		B	CGAAAGCCATGACCTCCGATCACTCTTTATAGCTTCTTCTACACCAACATCCAGGATGGACTTGGAAGGGTCCTT
*HPRT1*	NM 000194.1	A	TGAGCACACAGAGGGCTACAATGTGATGGCCTCCCATCTCCTTCATCACACACCGTGTGGACGGCAACTCAGAGATAACGCATAT
		B	CGAAAGCCATGACCTCCGATCACTCCAGTGCTTTGATGTAATCCAGCAGGTCAGCAAAGAATTTATAGCCCCCCT
*POLR2A*	NM 000937.2	A	ACTGGCCCAACAGGAAGACAGTAAGCGAAGGAGTCTTTGGCTTCTTGGAACCTGGAGTTTATGTATTGCCAACGAGTTTGTCTTT
		B	CGAAAGCCATGACCTCCGATCACTCCAGACGGCACAGAATATCCTTGGCTCTCTCAGCATCTCGAGCGG
*ACTB*	NM 001101.2	A	GATCTTGATCTTCATTGTGCTGGGTGCCAGGGCAGTGATCTCCTTCTGCACAGATAAGGTTGTTATTGTGGAGGATGTTACTACA
		B	CGAAAGCCATGACCTCCGATCACTCAGGATGGAGCCGCCGATCCACACGGAGTACTTGCGCTCAGGAGGAGCAAT

**Table III:** Signature IFN-II probes used for ISG and housekeeping gene quantification with NanoString technology

Gene	Accession no.	Probe	Sequence (5′-3′)
*ANKRD22*	NM 144590.2	A	ATGTTACAACCCTGAGGAGCCGGTTTCTGAGTCATATTTGCTCTGCCAGTCATAAAATTGGTTTTGCCTTTCAGCAATTCAACTT
		B	CGAAAGCCATGACCTCCGATCACTCGAAACAAAGGCACTGGTGTCATGTGTAACGACCCAAGCCTGTATCATCTA
*FCGR1B*	NM 001017986.3	A	GTAGGTGCCATTGTGACTTATGTTGGTTTTCAGAATGGTGAGGTTAGAATCCTGCCAATGCACTCGATCTTGTCATTTTTTTGCG
		B	CGAAAGCCATGACCTCCGATCACTCTGTGATATTCCTGCTGATGTGTAGCGATGCTTTCCCATGCCTGAGCAATG
*HLA-DMB*	NM 002118.3	A	TGGGGATACCCAGCCCCTAGATATTAAATCTGTTCCTTCCAGCTCACGCAACAGCCACTTTTTTTCCAAATTTTGCAAGAGCC
		B	CGAAAGCCATGACCTCCGATCACTCGAGCAGAATACTATATTGCCCGGGTCCCTTGACCCCCCAAATGAGTGATG
*HLA-DPB1*	NM 002121.4	A	GATAGAAACTGACTTCAGAGCAACTTCTTGGCAGCAGTATCCAATTTGGACGAACCTAACTCCTCGCTACATTCCTATTGTTTTC
		B	CGAAAGCCATGACCTCCGATCACTCTGGTTGGAGGCCCAGTGAGAGAAACAGTGCTTTGAATCAAAGAGCAGAAT
*HLA-DRB3*	NM 022555.4	A	ACAGCCCGGCCCCAAGGAAGAGCAGGCCCAGCACAAAGCCCCCGACTCCTTTCGGGTTATATCTATCATTTACTTGACACCCT
		B	CGAAAGCCATGACCTCCGATCACTCTCCTGTTGGCTGAAGTCCAGAGTGTCCTTTCTGATTCCTGAAGTAGATGA
*HPRT1*	NM 000194.1	A	TGAGCACACAGAGGGCTACAATGTGATGGCCTCCCATCTCCTTCATCACACACCGTGTGGACGGCAACTCAGAGATAACGCATAT
		B	CGAAAGCCATGACCTCCGATCACTCCAGTGCTTTGATGTAATCCAGCAGGTCAGCAAAGAATTTATAGCCCCCCT
*POLR2A*	NM 000937.2	A	ACTGGCCCAACAGGAAGACAGTAAGCGAAGGAGTCTTTGGCTTCTTGGAACCTGGAGTTTATGTATTGCCAACGAGTTTGTCTTT
		B	CGAAAGCCATGACCTCCGATCACTCCAGACGGCACAGAATATCCTTGGCTCTCTCAGCATCTCGAGCGG
*ACTB*	NM 001101.2	A	GATCTTGATCTTCATTGTGCTGGGTGCCAGGGCAGTGATCTCCTTCTGCACAGATAAGGTTGTTATTGTGGAGGATGTTACTACA
		B	CGAAAGCCATGACCTCCGATCACTCAGGATGGAGCCGCCGATCCACACGGAGTACTTGCGCTCAGGAGGAGCAAT

#### IFN-I and IFN-II scores

For both scores, the absolute count of each ISG was normalized by the geometric mean of the counts from 3 housekeeping genes: *ACT* (β-actin), *HPRT1* (hypoxanthine phosphoribosyltransferase 1), and *POLR2A* (RNA polymerase II subunit A). Relative expression was determined by dividing the normalized expression of each ISG by the median of its expression in 34 HDs (for the IFN-I score) or 17 HDs (for the IFN-II score). Finally, the median relative expression of these 6 and 5 ISGs constituted the IFN-I score and IFN-II score, respectively.

### IFN-α2 Simoa

IFN-α2 concentrations were determined on a Simoa HD-1 Analyzer according to the manufacturer’s instructions (IFN-α kit 100860, Quanterix, Billerica, Mass). Detailed information on the Simoa HD-1 Analyzer has been previously reported.^29^ Samples were measured either in duplicate or as single measurements. The detection threshold and lower limit of quantification of this assay were, respectively, 3.2 and 16 fg/mL.

### Cytokine stimulation

Whole blood cells were stimulated with 40,000 IU/mL of human IFN-α2 or IFN-γ (Miltenyi Biotec, San Diego, Calif) for 24 hours at 37°C + 5% CO_2_.

### Statistical analyses

Correlations were assessed by the Spearman rank correlation coefficient. The association between IFN-α2 measurements and IFN-I score were analyzed by a tobit regression model (to consider the quantification threshold) by quadratic polynomials. For the analysis of the area under the receiver operating characteristic (ROC) curve, samples with an IFN-α2 concentration between the detection threshold and the lower limit of quantification were also included. Statistical comparisons were performed by the Kruskal-Wallis test and the Wilcoxon-Mann-Whitney *U* test when appropriate. *P* < .05 was considered statistically significant.

## Results

### Expression of *IFI27, IFI44L,* and *SIGLEC1* is tightly related to IFN-α2 concentration

**Fig 1:**
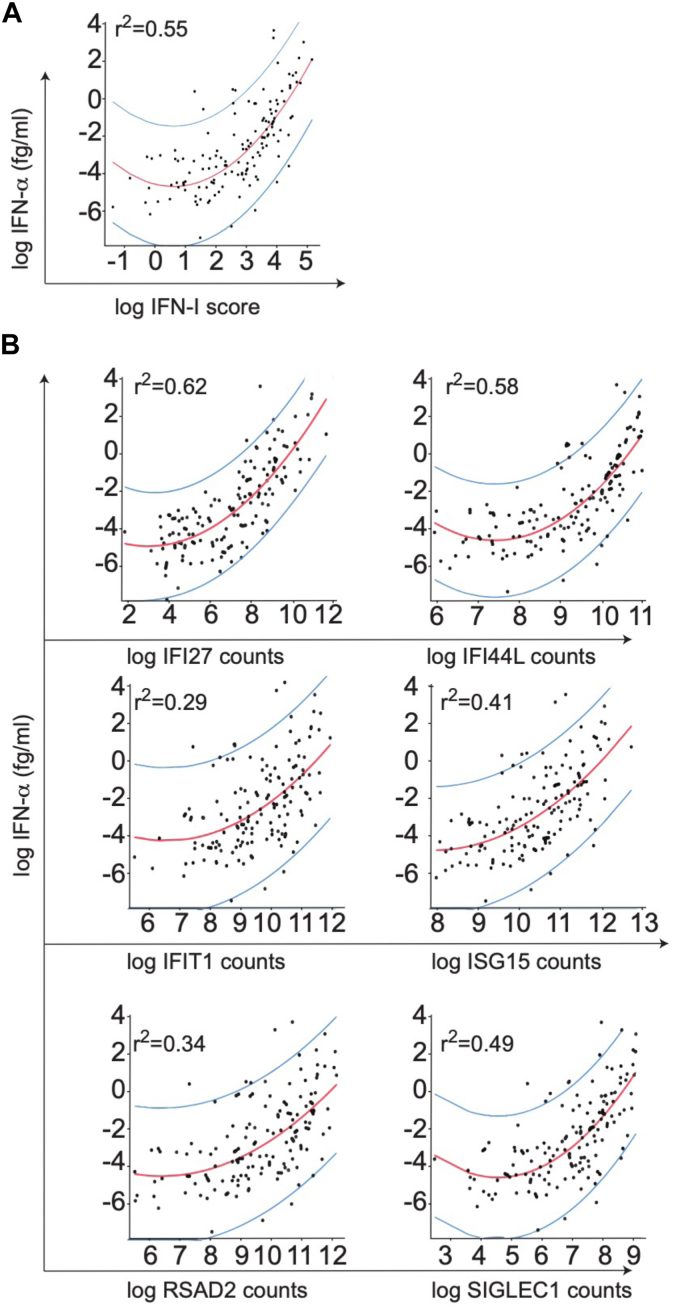
*IFI27, IFI44L,* and *SIGLEC1* expressions are most closely related to IFN-α concentration. We analyzed 196 samples from 119 patients by NanoString technology and Simoa. **(A)** Association between IFN-α2 protein levels and IFN-I score was assessed by Spearman rank correlation coefficient and analyzed by tobit regression model by quadratic polynomials. **(B)** Association between level of expression of each of 6 interferon-stimulated genes composing IFN-I score and IFN-α2 protein concentration.

We have previously described a NanoString-based protocol for measuring the expression of several ISGs *(IFI27, IFI44L IFIT1, SIGLEC1, ISG15, RSAD2)* and calculating, based on this, an IFN-I score. This score is elevated in patients with type I interferonopathies.^25^ We first compared the IFN-I score obtained with this NanoString-based protocol and the IFN-α2 levels measured by the Simoa technology across 196 samples collected from 119 pediatric patients with diverse clinical conditions (SLE = 22, DM = 7, mCTD = 9, monogenic type I interferonopathies [Aicardi-Goutières syndrome] = 4, other diseases = 77) and 7 HDs. The demographic and clinical-biological characteristics of the patients are provided in Table I. We observed a nonlinear relationship between IFN-α2 measurements and IFN-I score, and this analysis revealed an *r*^2^ coefficient of 0.55 (Fig 1, *A*). When we separately analyzed the relative expression of the 6 ISGs, the *r*^2^ coefficients obtained ranged from 0.29 to 0.62 and identified *IFI27, IFI44L,* and *SIGLEC1* as the ISGs most closely associated with plasma IFN-α2 concentration (*r*^2^ = 0.62, 0.58, and 0.49, respectively) and *IFIT1* the least well correlated (*r*^2^ = 0.29) (Fig 1, *B*).

### NanoString-based IFN-I score and Simoa IFN-α2 show comparable diagnostic performance of IFN-I–mediated diseases in children

**Fig 2:**
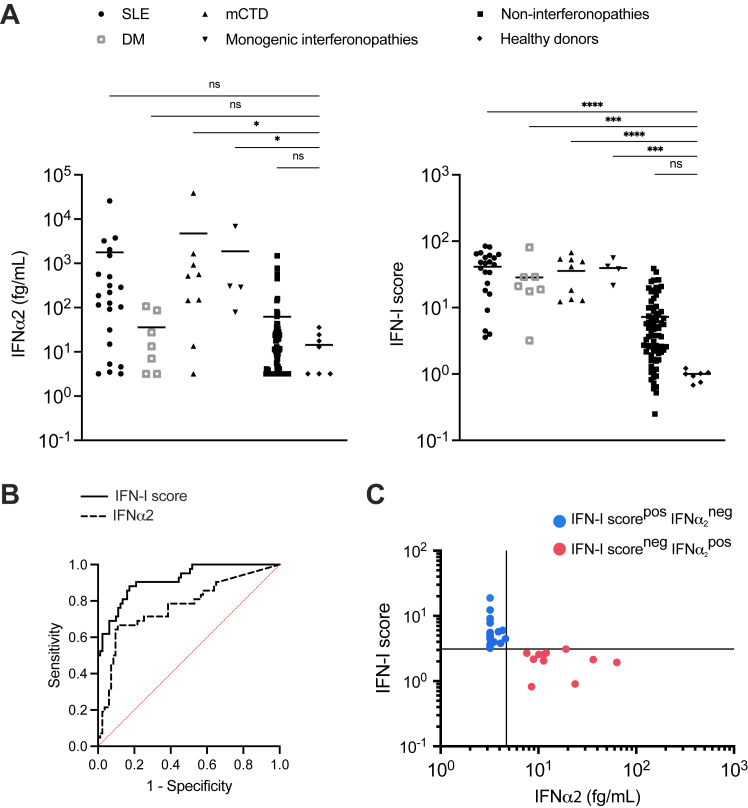
Comparison of diagnostic performance of NanoString and Simoa IFN-α2. Samples from 116 patients with either interferonopathies (22 SLE, 7 DM, 4 monogenic type I interferonopathies, 9 other connective tissue diseases) or other diseases (n = 74) and 7 healthy controls were analyzed for Simoa IFN-α2 and IFN-I signature by NanoString. **(A)** IFN-α2 concentration (fg/mL) and IFN-I score (log) plotted for each sample by disease. Statistical analysis was performed by Kruskal-Wallis test (*ns,* not significant; ∗*P* < .05, ∗∗∗*P* < .001, ∗∗∗∗*P* < .0001). *Black horizontal lines* represent mean for each group. **(B)** Analysis of ROC curve of IFN-I score and plasma IFN-α2. **(C)** NanoString and IFN-α2 Simoa were performed on 116 samples and interpreted with 3.1 and 4.7 fg/mL used as thresholds for IFN-I score and IFN-α2 Simoa, respectively. Thirty discordant results were identified between these two techniques: 11 IFN-I score^neg^IFN-α2^pos^ samples *(red dots)* and 19 IFN-I score^pos^IFN-α2^neg^ samples *(blue dots)*. *IFNopathies,* Interferonopathies.

To assess diagnostic performance, we retained only one sample per patient (the oldest) and excluded 3 patients from the “other diseases” group who had influenza A virus infection, a condition known to activate the IFN-I pathway, who did not meet the criteria for this group but whose samples were too few to permit retention as a separate group. We first analyzed the results by disease group. As depicted in Fig 2, *A,* we observed a significant upregulation of IFN-α2 at the protein level in patients with mCTDs (*P* = .0405) and monogenic type I interferonopathies (*P* = .0498) compared to HDs. IFN-α2 concentration was also higher in SLE patients compared to HDs, but the difference was not significant. IFN-I score was significantly higher in these groups (*P* < .0001 for mCTDs and *P* = .0002 for monogenic type I interferonopathies) but also in SLE patients (*P* < .0001) and in DM patients (*P* = .0003), but not in patients in the “other diseases” group (*P* = .0503), compared to HDs.

Analysis of the area under the ROC curve (95% confidence interval) showed that both IFN-α2 concentration (area under the curve = 0.771) and IFN-I score (area under the curve = 0.917) robustly discriminate type I interferonopathies and IFN-I–related autoimmune diseases from others with an advantage of the transcriptomic approach (*P* < .001) (Fig 2, *B*). Analysis of the ROC curve also allowed us to define the optimal thresholds for each assay, namely 3.1 for the IFN-I score (sensitivity = 100%, specificity = 48%) and 4.7 fg/mL for IFN-α2 quantification (sensitivity 83%, specificity 42%). Using these thresholds, among the 116 samples tested, we identified 30 discordant results between the two techniques, including 11 samples with IFN-I score of <3.1 and IFN-α2 of >4.7 fg/mL (IFN-I score^neg^IFN-α2^pos^ samples, IFN-α2 concentration ranging from 7.60 to 63.30 fg/mL) and 19 with IFN-I score of >3.1 and IFN-α2 of <4.7 fg/mL (IFN-I score^pos^IFN-α2^neg^ samples, IFN-I score ranging from 3.19 to 18.81) (Fig 2, *C*).

### IFN-I and IFN-II induce different transcriptomic signatures that can be monitored by NanoString technology

**Fig 3:**
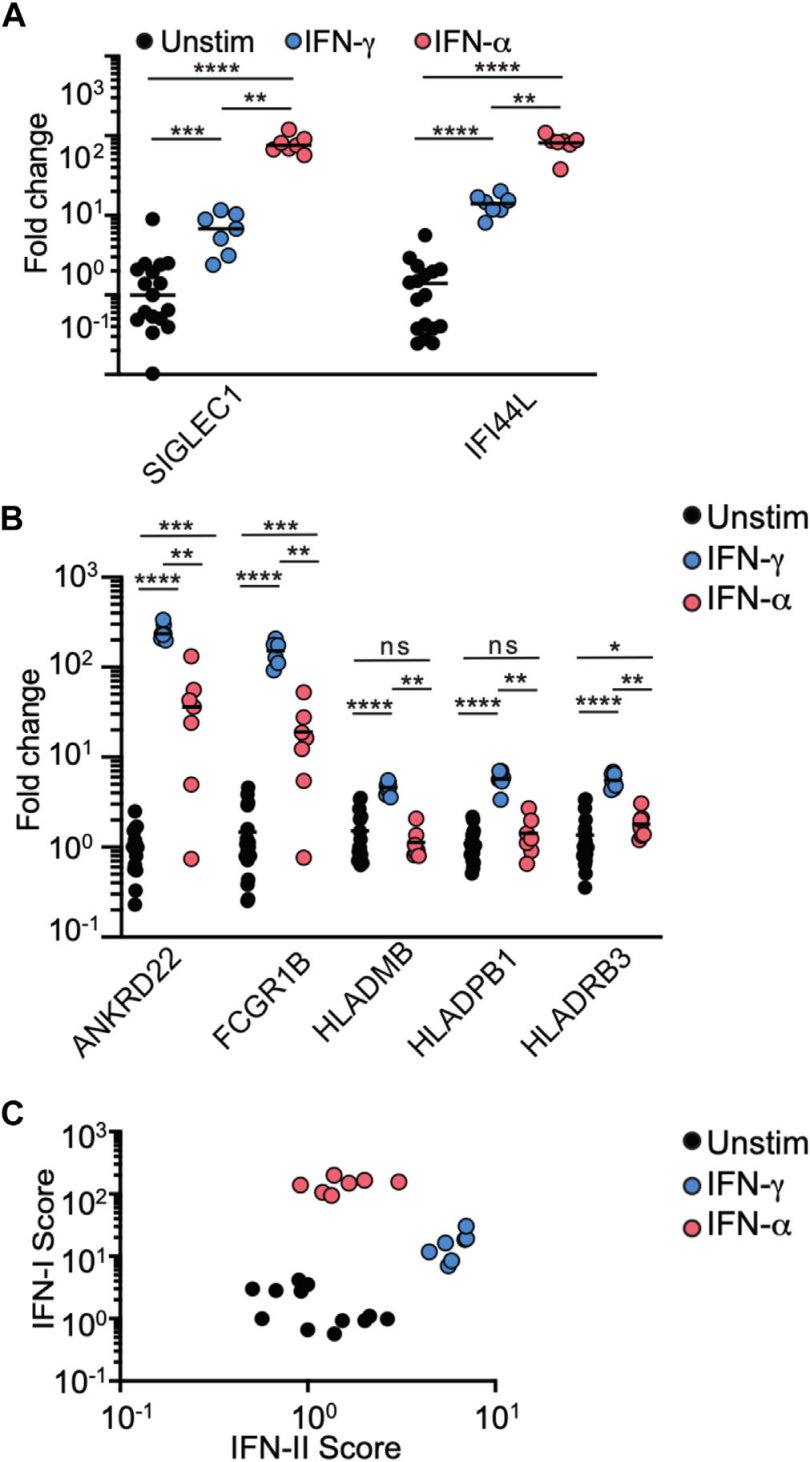
IFN-I and IFN-II induce distinct transcriptomic signatures identified with NanoString technology. **(A)** Fold change increase of expression of two ISGs composing IFN-I score *(SIGLEC1, IFI44L)* after stimulation of whole blood cells with either IFN-γ or IFN-α2. **(B)** Fold change increase of expression of 5 previously identified IFN-γ–inducible genes *(ANKRD22, FCGR1B, HLA-DMB, HLA-DPB1, HLA-DRB3)* after stimulation of whole blood cells with either IFN-γ *(blue dots)* or IFN-α2 *(red dots)*. **(C)** Coanalysis of IFN-I score and IFN-II score after IFN-γ *(blue dots)* or IFN-α2 *(red dots)* stimulation. IFN-II score was calculated as median of 5 IFN-γ–inducible genes’ relative expression compared to healthy controls. Statistical analysis was performed by Kruskal-Wallis test (*ns,* not significant; ∗*P* < .05, ∗∗*P* < .01, ∗∗∗*P* < .001, ∗∗∗∗*P* < .0001).

Next, we investigated the underlying reasons for the disparities between IFN-I score and IFN-α2 concentration in certain patients. Many ISGs are induced by both IFN-I and IFN-II, and our group has previously demonstrated that IFN-γ can lead to a low positive IFN-I score *in vitro.*^25^ We hypothesized that other interferons, including IFN-γ, might in part explain the disparities between IFN-I score and IFN-α2 concentration in certain patients. To substantiate this point, we compared the expression of 2 ISGs strongly correlated with IFN-α2 concentration (*SIGLEC1* and *IFI44L*) in whole blood cells after stimulation with IFN-α or IFN-γ *in vitro.* Both IFN-α and IFN-γ increased *SIGLEC1* and *IFI44L* expression, but this increase was about 10 times lower with IFN-γ (Fig 3, *A*). Therefore, IFN-I and IFN-II induce the expression of identical ISGs but at different levels. Yet on the basis of their expression levels, the ISGs composing the IFN-I score appear more specific to IFN-I than to IFN-II.

In order to discriminate between the effects of type I and type II interferons, we sought to develop a transcriptomic signature specific of the IFN-II pathway. To this end, we analyzed the expression levels of 5 IFN-γ-inducible genes *(ANKRD22, FCGR1B, HLA-DMB, HLA-DPB1, HLA-DRB3)* at steady state or in response to IFN-γ or IFN-α2 stimulation *in vitro.* The expression levels of *ANKRD22*, *FCGR1B,* and *HLA-DRB3* were significantly increased after IFN-γ and IFN-α stimulation compared to unstimulated samples but were significantly higher after IFN-γ stimulation. The expression of *HLA-DMB* and *HLA-DPB1* increased exclusively in response to IFN-γ stimulation (Fig 3, *B*). Overall, these results confirmed the specificity of these 5 ISGs for IFN-γ, and their expression levels were used to calculate an IFN-II score corresponding to the median fold change of expression of these 5 ISGs.

To assess the ability of the two interferon scores to distinguish between IFN-I and IFN-II responses, we stimulated blood samples with IFN-γ or IFN-α2 and compared the two scores for each stimulated condition. As expected, IFN-γ exposure led to a high IFN-II score and low IFN-I score, while IFN-α2 stimulation led to a high IFN-I score and low IFN-II score (Fig 3, *C*). These results confirmed that different transcriptomic signatures are induced by IFN-I or IFN-II and can be monitored by the NanoString technology.

### The combined analysis of IFN-I and IFN-II scores defines subgroups of patients

**Fig 4:**
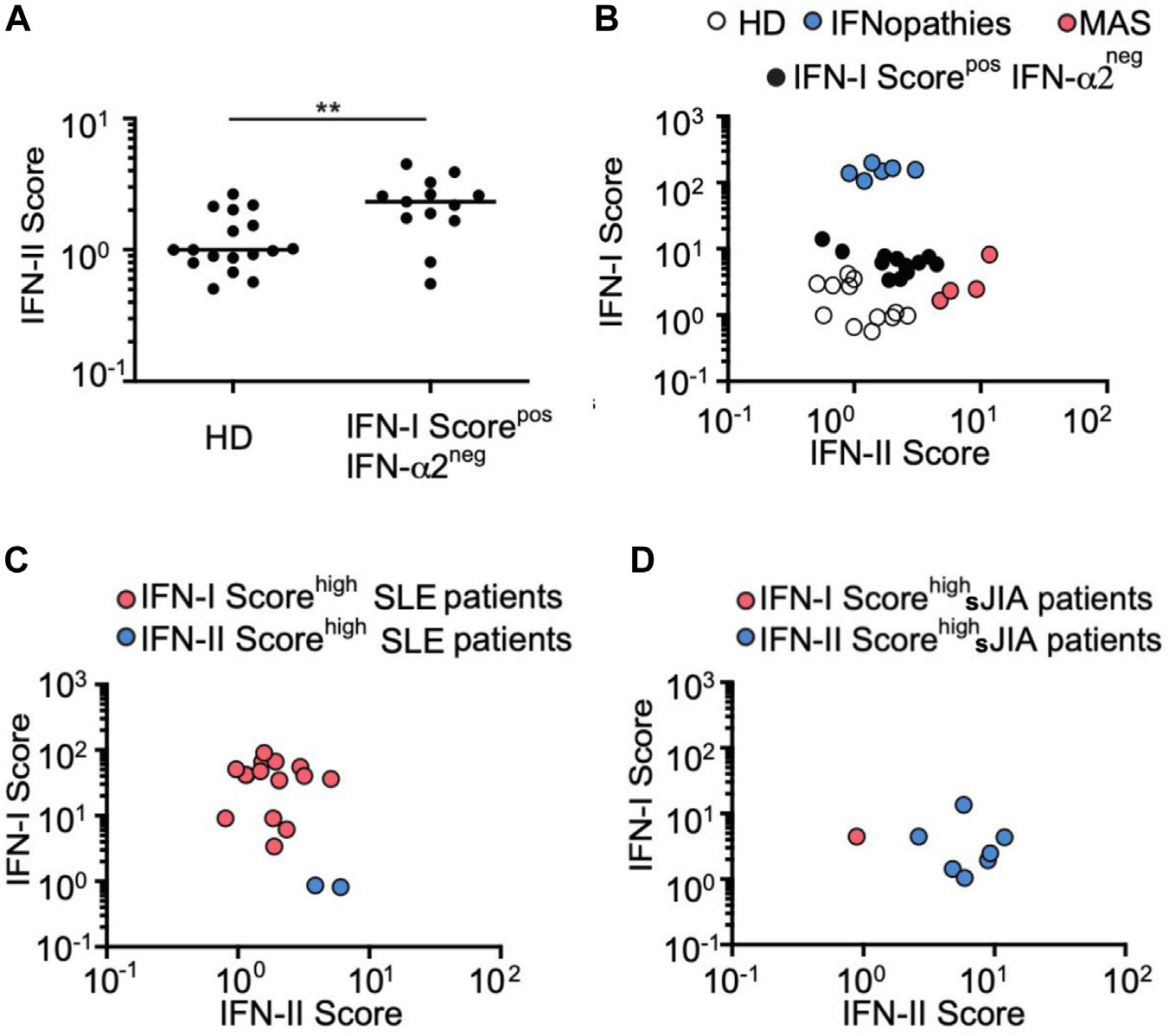
Concomitant analysis of IFN-I score and IFN-II score reveals different transcriptomic profiles within single disease. **(A)** IFN-II score calculated for 13 discordant IFN-I score^pos^IFN-α2^neg^ samples. Statistical analysis was performed by Wilcoxon-Mann-Whitney *U* test (∗∗*P* < .01). *Black horizontal lines* represent mean for each group. **(B)** Coanalysis of IFN-I score and IFN-II score of patients with monogenic type I interferonopathies (n = 6, *blue dots*), patients with MAS (n = 4, *red dots*), and 13 patients with IFN-I score^pos^IFN-α2^neg^*(black dots).***(C)** Coanalysis of IFN-I score and IFN-II score of patients with SLE (n = 16). **(D)** Coanalysis of IFN-I score and IFN-II score of patients with sJIA (n = 8).

We then aimed to determine the role of IFN-γ in the divergent results observed for 19 samples (IFN-I score^pos^IFN-α2^neg^). Thirteen of these samples were analyzed by the assays described above. We observed a significant increase in IFN-II score (*P* = .0034) in those samples compared to HDs, thus explaining the discrepancy between IFN-I score and IFN-α2 concentration (Fig 4, *A*).

We then compared the IFN-I (recalculated based only on the expression of *SIGLEC1* and *IFI44L*) and IFN-II scores for 6 patients with monogenic type I interferonopathies, 4 with MAS, and 12 HDs. MAS is a life-threatening complication of several systemic inflammatory diseases in which increased serum IFN-γ levels are described.^30^ For each group, the samples grouped together, at a distance from the two other groups. When we plotted the IFN-I score^pos^IFN-α2^neg^ samples on this graph, we observed that 11 clustered in close proximity to MAS, providing compelling evidence for the contribution of IFN-γ in the upregulation of ISGs for this disease (Fig 4, *B*). We also calculated IFN-I and IFN-II scores for 16 patients with SLE, a disease mainly known to be mediated by IFN-I, and for 8 patients with sJIA. The physiopathology of sJIA remains scarcely known, but proinflammatory cytokines are abundant in the serum and synovial fluid of sJIA patients, including IFN-γ–derived chemokines (CXCL10, CXCL9).^31^ By analyzing the level of each interferon score on the same graph, we were able to distinguish 2 groups among SLE patients: 14 IFN-I score^high^IFN-II score^low^ samples and 2 IFN-I score^low^IFN-II score^high^ samples (Fig 4, *C*). In sJIA patients, two groups were also identified according to IFN-II score: 7 IFN-II score^high^ and 1 IFN-II score^low^IFN-I score^high^ (Fig 4, *D*). These data demonstrate that within a group of patients presenting with the same disease, the concomitant analysis of the two interferon scores makes it possible to distinguish subgroups of patients for whom pathogenic mechanisms could be different.

## Discussion

Two main approaches are used to monitor IFN-I pathway activation: ISG expression signatures and direct plasma IFN-α quantification. Despite the several technologies that have been developed, few are validated for routine laboratory use, and no consensus exists on the preferred method for routine care. The European Alliance of Associations for Rheumatology recently issued recommendations to address this lack of standardization.^18^^,^^23^

ISG mRNA expression can be analyzed by real-time reverse transcription–quantitative PCR (RT-qPCR) or the NCounter platform, but ISG selection and preanalytical conditions may affect diagnostic accuracy.^20^^,^^21^^,^^25^^,^^32^^,^^33^ Similarly, digital ELISA (Simoa) is widely used, but the use of homemade versus commercial reagents may cause interlaboratory variability.^9^^,^^10^^,^^32^ These discrepancies make comparing results between laboratories difficult. Given the significance of circulating IFN-α levels as a reliable disease activity biomarker,^9^, ^10^, ^11^, ^12^^,^^14^ standardizing interferon pathway assays is crucial for diagnosis and follow-up of patients. Previous studies reported high agreement between IFN-α quantification and ISG scores, but to our knowledge, most involved only adult cohorts or used in-house protocols or RT-qPCR.^9^^,^^14^^,^^32^ Rodero and Crow^9^ were the first to compare the two approaches but by homemade protocols, and a recent study comparing IFN-α Simoa and IFN-I score by RT-qPCR in pediatric SLE showed a positive correlation between the two assays.^14^

Here we report the comparison between an IFN-I score based on the expression of 6 ISGs measured by NanoString technology and circulating IFN-α2 concentration by using Simoa and its commercial reagents in a large cohort of 119 pediatric patients, including 42 patients with IFN-I–mediated diseases. The two methods correlated, and we identified *IFI27, IFI44L,* and *SIGLEC1* as the ISGs most significantly associated with IFN-α2 concentration. These results are consistent with recent findings suggesting that a reduced set of genes, namely *IFI27* and *IFI44L,* may serve as markers of systemic IFN-I activation.^34^ Both Simoa plasma IFN-α2 and IFN-I score distinguished IFN-I–related disease from HDs (Fig 2, *A* and *B*), in agreement with previous findings comparing the interferon signature (NanoString) and IFN-α2 quantification (Simoa) in a large cohort of adult SLE patients.^32^ However, the correlation coefficients were not very high, and only the IFN-I score was able to distinguish all IFN-I–mediated diseases from HDs (Fig 2, *A*). Variations in disease activity, different patient treatments, and the effect of other interferons on the IFN-I score, particularly IFN-γ, which we explored later, could partially explain these differences. Another limitation of this study is the small sample size in certain groups, such as type I monogenic interferonopathies and DM, which limits our ability to draw definitive conclusions about the performance of the tests in these groups.

It has been shown that IFN-I and IFN-II induce overlapping ISGs expression.^2^^,^^3^^,^^25^ Here, IFN-γ exposure also induced the transcription of ISGs triggered by IFN-α, although at lower levels, thereby highlighting pathway overlaps. This may explain discrepancies in patients with IFN-I score–positive but IFN-α2–negative results. Notably, the Simoa assay only measures IFN-α2 concentration, excluding contribution of other IFN-I, including other IFN-α and IFN-β. The involvement of other interferons (other IFN-I and IFN-III) was not evaluated but could certainly provide new information to further characterize interferon pathway activation in these diseases. Nevertheless, these results suggest that our IFN-I score reflects both IFN-I and, to a lesser extent, IFN-II pathway activation. As for the clinical implications of these findings, on the one hand, Simoa may be better suited to monitor therapies targeting IFN-α rather than using the IFN-I score, which is affected by other signals. On the other hand, IFN-I score assessed by NanoString technology seems perfectly suited (and is easier to use than Simoa) for monitoring treatments targeting both IFN-I and IFN-II signaling pathways (eg, JAK inhibitors).

The overlap between IFN-I and IFN-II signaling did not explain the discordant IFN-I score^neg^IFN-α2^pos^ samples. On closer examination of these samples, only 4 patients had an IFN-α2 concentration higher than 16 fg/mL, and all were from the “other diseases” group (Castleman disease, Kabuki syndrome, urticaria, and idiopathic thrombocytopenic purpura). Two patients were treated with glucocorticoids, which have been reported to suppress ISG expression.^35^ Thus, although it does not explain all the discrepancies, one hypothesis is that the IFN-I signature was turned off by glucocorticoids while IFN-α2 was still detectable but probably no longer functional. This highlights another potential advantage of using the IFN-I signature over IFN-α2 quantification with Simoa or combining both techniques.

To specifically assess IFN-γ pathway activation, we designed an IFN-II score quantifying the expression of 5 IFN-γ-inducible genes using the NanoString-based method. *HLA-DMB* and *HLA-DPB1* were specifically upregulated by IFN-γ. Future studies should assess the influence of other factors, including other IFN-I subtypes and IFN-III, on these genes before considering the clinical implementation of the IFN-II score. We confirmed that IFN-I and IFN-II induced distinct transcriptomic signatures, and we found a significant increase of IFN-II score in the IFN-I score^pos^IFN-α2^neg^ samples, suggesting high IFN-γ levels in these patients. Combined analysis of IFN-I score and IFN-II score allowed discrimination of monogenic type I interferonopathies from diseases associated with increased IFN-II pathway activation (MAS). These scores also identified distinct transcriptomic profiles defining subgroups within the same disease (ie, SLE and sJIA), albeit on a small number of patients. These findings support developing a single assay, instead of 2 or 3 (IFN-I score, IFN-α, IFN-γ), to monitor both IFN-I and IFN-II pathways—for example, measuring the relative expression of *IFI27, IFI44L,* and *SIGLEC1* (IFN-I) and *HLA-DMB* and *HLA-DPB1* (IFN-II). Such an assay could simplify diagnostics and allow better stratification of patients, as suggested by prior studies by using IFN-I– and IFN-II–stimulated gene expression scores to stratify inflammatory and dysimmune myopathies.^26^

Beyond diagnosis, the IFN-I score can monitor disease activity (DM) and assess interferon-targeted therapies efficacy (eg, JAK inhibitors, anti-IFNAR antibodies). As cytokine-targeting therapies are increasingly used in the treatment of dysimmune diseases,^15^^,^^16^^,^^36^ reliable monitoring tools are essential. Transcriptomic approaches like ISG scores are advantageous because of the difficulty of detecting low cytokine levels in serum. These scores have shown utility as biomarkers of disease activity and treatment response, such as for JAK inhibitors in DM or haploinsufficiency of A20.^15^^,^^16^^,^^37^^,^^38^ An IFN-I/IFN-II score could guide therapeutic decision-making and help monitoring treatments targeting IFN-α, IFN-γ, or both (anifrolumab, emapalumab, JAK inhibitors). However, the evolution of the score in response to treatment requires further evaluation. Beyond interferons, mRNA-based assays may also be useful to monitor exposure to other cytokines (TNF-α, IL-1, IL-6), which are common drug targets in inflammatory conditions.^39^, ^40^, ^41^

In conclusion, both IFN-α2 quantification by Simoa and the NanoString-based IFN-I score reliably distinguish type I interferonopathies from other diseases in pediatric patients, although some discrepancies remain. IFN-II likely contributes to these differences. Additional factors, including disease activity, treatments, and analytical considerations (eg, measurement of plasma proteins vs quantification of RNA in circulating cells) should also be taken into account. IFN-I and IFN-II induce different transcriptomic signatures *in vitro* and *in vivo* that can be distinguished by the NanoString technology. By designing a type II ISG score, we showed that the IFN-I score and the IFN-II score discriminate subgroups of patients with the same disease, underscoring the potential of such tools for precision diagnostics and personalized medicine to guide treatment decisions in autoinflammatory diseases, particularly when choosing therapies that specifically target IFN-I (anti-IFNAR) versus those that target all types of interferon (JAK inhibitors).

## Disclosure statement

Supported by the 10.13039/501100006451Hospices Civils of Lyon (AO PAM BAP ACP).

Disclosure of potential conflict of interest: The authors declare that they have no relevant conflicts of interest.

## Supplementary data

Supplementary Table E1
