# Supplementary material for: Profiling type I and II interferon responses reveals distinct subgroups of pediatric patients with autoinflammatory disorders
Source: J Allergy Clin Immunol Glob. 2025 Mar 8;4(2):100450. doi: 10.1016/j.jacig.2025.100450 (PMC12002218; doi:10.1016/j.jacig.2025.100450)
Supplement: Supplementary Table E1 [file mmc1.docx]

*ANKRD22 ATF3 BATF2 CD274 CIITA ETV7 FCGR1B GBP1 GBP2 GBP5 HLA-DMB HLA-DOB HLA-DPB1*

*HLA-DRB3 IFI44L IFNb1 IFNψ*

*"IP10 (CXCL10)" PDK4 SERPING1 SIGLEC1*

**Table S1.** List of the 21 genes analyzed using the Nanostring technology, in whole blood samples at basal state or following a 24-hour stimulation with IFNα or IFNψ.
